# Abscisic acid (ABA) regulates grape bud dormancy, and dormancy release stimuli may act through modification of ABA metabolism

**DOI:** 10.1093/jxb/eru519

**Published:** 2015-01-05

**Authors:** Chuanlin Zheng, Tamar Halaly, Atiako Kwame Acheampong, Yumiko Takebayashi, Yusuke Jikumaru, Yuji Kamiya, Etti Or

**Affiliations:** 1 ^1^ Institute of Plant Sciences, Department of Fruit Tree Sciences, Agricultural Research Organization, Volcani Center, Bet Dagan 50250, Israel; 2 ^2^ Institute of Plant Sciences and Genetics in Agriculture, The Faculty of Agriculture, Food and Environment, The Hebrew University of Jerusalem, Rehovot 76100, Israel; 3 ^3^ RIKEN Plant Science Center, Yokohama, Kanagawa 230-0045, Japan

**Keywords:** ABA 8′-hydroxylase, abscisic acid, bud, 9-*cis*-epoxycarotenoid dioxygenase, dormancy, grapevine.

## Abstract

In warm-winter regions, induction of dormancy release by hydrogen cyanamide (HC) is mandatory for commercial table grape production. Induction of respiratory stress by HC leads to dormancy release via an uncharacterized biochemical cascade that could reveal the mechanism underlying this phenomenon. Previous studies proposed a central role for abscisic acid (ABA) in the repression of bud meristem activity, and suggested its removal as a critical step in the HC-induced cascade. In the current study, support for these assumptions was sought. The data show that ABA indeed inhibits dormancy release in grape (*Vitis vinifera*) buds and attenuates the advancing effect of HC. However, HC-dependent recovery was detected, and was affected by dormancy status. HC reduced *VvXERICO* and *VvNCED* transcript levels and induced levels of *VvABA8’OH* homologues. Regulation of these central players in ABA metabolism correlated with decreased ABA and increased ABA catabolite levels in HC-treated buds. Interestingly, an inhibitor of ethylene signalling attenuated these effects of HC on ABA metabolism. HC also modulated the expression of ABA signalling regulators, in a manner that supports a decreased ABA level and response. Taken together, the data support HC-induced removal of ABA-mediated repression via regulation of ABA metabolism and signalling. Expression profiling during the natural dormancy cycle revealed that at maximal dormancy, the HC-regulated *VvNCED1* transcript level starts to drop. In parallel, levels of *VvA8H-CYP707A4* transcript and ABA catabolites increase sharply. This may provide initial support for the involvement of ABA metabolism also in the execution of natural dormancy.

## Highlight

ABA has a central role in repression of grape bud meristem activity, and both the natural dormancy cycle and artificial dormancy release stimuli act via regulation of ABA metabolism.

## Introduction

In warm-winter regions, dormancy release poses a major obstacle to commercial viticulture. Artificial substitutes for chilling are thus mandatory in these regions to avoid prolonged dormancy, thereby allowing co-ordinated and early production of economically viable yields. The only practical means currently available for effective artificial dormancy release in vineyards involves treatment with hydrogen cyanamide (HC), used by the table grape industry worldwide (Lavee and May, 1997; Or, 2009). The ability of HC to induce respiratory stress, which initiates a biochemical cascade that leads to effective dormancy release, is also responsible for its toxicity, both to the vines and to the environment (Ophir *et al.*, 2009; Or, 2009; Pérez *et al.*, 2009; Vergara *et al.*, 2012). Development of safe alternatives may rely on the manipulation of targets that are affected by the artificial stimuli downstream of the respiratory stress, which stands a much better chance of being plant specific and harmless. A detailed characterization of such targets is currently unavailable.

The results of a large-scale comparative analysis of grape (*Vitis vinifera*) bud responses to two artificial stimuli of bud dormancy release, HC and heat shock (HS), allowed a working model of the events occurring during artificially induced bud dormancy release to be proposed (Ophir *et al.*, 2009) (Supplementary Fig. S1 available at *JXB* online). According to this model, perturbation of cytochrome pathway activity in the mitochondria leads to respiratory and oxidative stress, expressed as an increased level of reactive oxygen species, decreased activity of the tricarboxylic acid cycle, and decreased production of ATP. To address this energy crisis, the alternative oxidase pathway, glycolysis, pyruvate metabolism, and anaerobic respiration are induced, in an order that has yet to be defined. In parallel, the cellular antioxidant machinery and related pathways are up-regulated to cope with the oxidative burst. Changes resulting from the above reprogramming under conditions that mimic hypoxia may affect the interplay between ethylene and abscisic acid (ABA) in a way that allows removal of ABA repression of meristem activity and growth resumption. This hypothesis was inspired by similar scenarios played out in deepwater rice and *Rumex palustris* under low oxygen conditions (Benschop *et al.*, 2006; Steffens *et al.*, 2006; Lasanthi-Kudahettige *et al.*, 2007; Hattori *et al.*, 2009), and, as recently observed, during seed dormancy release as detailed below (Linkies *et al.*, 2009; Arc *et al.*, 2013).

The results of subsequent analyses supported the predictive power of the model: treatment with sodium azide (AZ), a well-known inhibitor of mitochondrial respiration, stimulated bud dormancy release in a manner similar to HC, and treatment with HC, a well-known dormancy release agent, inhibited O_2_ uptake by isolated grape bud mitochondria (Ophir *et al.*, 2009; Pérez *et al.*, 2009). Treatment with HC induced a temporary increase in hydrogen peroxide levels (Pérez *et al.*, 2008) and alternative oxidase transcripts (Ophir *et al.*, 2009). HC and HS transiently up-regulated various oxidative stress-related genes (Keilin *et al.*, 2007; Halaly *et al.*, 2008). HC and HS up-regulated expression of GDBRPK, a sucrose nonfermenting (SNF)-like protein kinase, which is a sensor of elevated AMP levels in stressful situations, as well as that of sucrose synthase (Halaly *et al.*, 2008), pyruvate decarboxylase, and alcohol dehydrogenase (Or *et al.*, 2000*a*; Keilin *et al.*, 2007; Halaly *et al.*, 2008; Ophir *et al.*, 2009). Production of both acetaldehyde and ethanol was detected following the application of dormancy release stimuli such as HC, HS, and AZ (Ophir *et al.*, 2009), and hypoxic conditions induced dormancy release (Vergara *et al.*, 2012). Enhancement of bud break by HC was shown to be dependent on calcium signalling, and HC induced changes in the transcription and phosphorylation of regulators of calcium signalling (Pang *et al.*, 2007). Moreover, various dormancy release stimuli temporarily induced endogenous ethylene production, and exogenous ethylene stimulated dormancy release, whereas treatment with an inhibitor of ethylene signalling inhibited dormancy release (Ophir *et al.*, 2009) and eliminated the enhancing effect of HC, AZ, and HS (E. Or *et al.*, unpublished). In the current study, the model was further tested by investigating the hypothesis that ABA is involved in dormancy maintenance, and that HC stimulates the removal of this repression.

### ABA and seed dormancy

ABA produced by zygotic tissues at late maturation stages appears to be a central regulator of seed dormancy and germination, and modifications in its metabolism or signalling lead to significant dormancy-related phenotypes (Karssen *et al.*, 1983; Frey *et al.*, 2004; Arc *et al.*, 2013). In general, deficiency in ABA and its synthesis, as well as interference in ABA signalling, lead to dormancy loss, while suppression of ABA inactivation leads to increased depth of dormancy (Nambara and Marion-Poll, 2005; Nambara *et al.*, 2010).

Carotenoid cleavage by 9-*cis*-epoxycarotenoid dioxygenase (NCED) has been proven to constitute a key regulatory step in the control of ABA synthesis, which affects seed dormancy and germination (Iuchi *et al.*, 2001; Qin and Zeevaart, 2002; Cadman *et al.*, 2006; Lefebvre *et al.*, 2006). Accordingly, (i) an *Arabidopsis nced6nced9* double mutant exhibited reduced ABA content and reduced seed dormancy (Lefebvre *et al.*, 2006); (ii) overexpression of *NCED* increased the ABA level and dormancy in tomato seeds, and delayed germination in imbibed tobacco seeds (Qin and Zeevaart, 2002); and (iii) induction of *NCED* was sufficient to suppress germination of imbibed seeds despite their exposure to dormancy release treatment (Martínez-Andújar *et al.*, 2011). Additional ABA-deficient mutants with impaired synthesis, such as *aba1*, *aba2*, and *aao3*, lacked the primary dormancy associated with mature *Arabidopsis* seeds (Leon-Kloosterziel *et al.*, 1996; Finkelstein *et al.*, 2002; Himmelbach *et al.*, 2003), and overexpression of *XERICO*, another positive regulator of the ABA level, also resulted in repression of seed germination (Ko *et al.*, 2006).

An additional key regulatory step for the control of ABA levels appears to be ABA inactivation by its hydroxylation at the 8′ position, catalysed by CYP707A ABA 8′-hydroxylase (ABA8’OH) (Cutler and Krochko, 1999; Nambara and Marion-Poll, 2005; Cutler, 2007; Nambara *et al.*, 2010). The significant effect of this step on both the ABA level and seed dormancy is reflected by (i) increased ABA levels in dry and imbibed seeds of *Arabidopsis cyp707a2* mutants and their reduced germination (Okamoto *et al.*, 2006); and (ii) the higher ABA levels and increased dormancy of transgenic *ABA8′OH1* RNAi (RNA interference) barley grains (Gubler *et al.*, 2008).

ABA signalling is also important in the control of seed dormancy. The interaction between protein phosphatase 2C (PP2C) and SNF1-related protein kinase 2 (SnRK2), which negatively affect ABA signalling, is disrupted following binding of ABA to its *pyr/pyl* receptors and formation of ABA receptor–PP2C complexes. This allows activation of SnRK2, which then activates downstream transcription factors that induce ABA-responsive gene expression (Hubbard *et al.*, 2010). In agreement with the role suggested for ABA in seed dormancy, germination of a *pyr/pyl* sextuple mutant was highly insensitive to ABA, and the triple mutant *snrk2.2snrk2.6snrk2.3* also exhibited loss of dormancy (Fujii and Zhu, 2009; Nakashima *et al.*, 2009; Gonzalez-Guzman *et al.*, 2012). PP2C functions also regulate germination ability. Accordingly, germination of *pp2c* mutants was slower than in the wild type and was inhibited by very low ABA concentrations, in agreement with its negative role in ABA signalling (Kuhn *et al.*, 2006; Rubio *et al.*, 2009). Overexpression of *AtPP2CA*, however, resulted in significantly improved germination at ABA concentrations that completely inhibited wild-type seed germination (Kuhn *et al.*, 2006).

### ABA and bud dormancy

A role for ABA in the regulation of bud endodormancy has been discussed in the literature, and it has been suggested that ABA levels increase in the autumn and act as a signal of shorter day-length. This, in turn, hypothetically results in inhibition of cell proliferation and shoot growth, promotion of terminal bud set, and induction of endodormancy. Accordingly, after 3–4 weeks of short days, regulators of ABA biosynthesis (NCED3, ABA1, and ABA2) and ABA signal transduction components (PP2C, ABI1, AREB3, among others) were induced in poplar buds, and ABA levels in the apex peaked (Arora *et al.*, 2003; Rohde and Bhalerao, 2007; Ruttink *et al.*, 2007). ABA application accelerated growth cessation in seedlings of two birch tree ecotypes (Li *et al.*, 2003), ABA levels were highest in deeply dormant potato tubers (Korableva *et al.*, 1980; Biemelt *et al.*, 2000; Destefano-Beltrán *et al.*, 2006*a*), and endogenous ABA levels also increased during onset of grape bud dormancy (Düring and Bachmann, 1975; Koussa *et al.*, 1994; Or *et al.*, 2000*b*). Decreased levels of ABA were recorded in leafy spurge during the transition from endodormancy to ecodormancy (Horvath *et al.*, 2006).

Based on the above, a role for ABA in the regulation of dormancy maintenance and release was considered, and then questioned due to conflicting results in the limited number of reported studies. In support of ABA’s role, a decrease in endogenous ABA level preceded bud dormancy release in birch, grapevine, and potatoes (Koussa *et al.*, 1994; Or *et al.*, 2000*b*; Li *et al.*, 2004; Destefano-Beltrán *et al.*, 2006*a*), and delayed bud break was reported following ABA application in birch (Rinne *et al.*, 1994*a*), apple (Dutcher and Powell, 1972), kiwi fruit (Lionakis and Schwabe, 1984), and sour cherry (Mielke and Dennis, 1978). However, spring application of ABA on grapes had little effect on bud break (Hellman *et al.*, 2006), and the effect of chilling on the endogenous ABA level is not clear. In agreement with the suggested role of ABA, chilling-induced dormancy release of birch was accompanied by alterations in endogenous ABA levels (Li *et al.*, 2004). However, no clear effect of chilling on birch bud ABA content was detected in another study (Rinne *et al.*, 1994*a*). Among the findings that put ABA’s role in dormancy maintenance/release into question are the similar decline in ABA levels of chilled and non-chilled apple buds despite induction of dormancy release only in the chilled buds, and the higher ABA content in chilled cherry buds compared with non-chilled controls (Saure, 1985; Powell, 1987; Crabbe, 1994).

In potato, declining ABA content throughout the dormancy cycle was correlated with decreased expression of *NCED1/2* (Destefano-Beltrán *et al.*, 2006*a*), and treatment of microtubers with an ABA biosynthesis inhibitor shortened the dormancy period (Suttle and Hultstrand, 1994). The level of ABA8′OH expression in tubers was inversely correlated to ABA levels and positively correlated to bud break (Debast *et al.*, 2011). Nevertheless, application of ABA to dormant tubers had no marked effect, whereas treatment of non-dormant tubers only transiently inhibited sprout growth, suggesting that variations in ABA degradation ability may play a central role in bud behaviour (Suttle *et al.*, 2012). An ~2-fold increase in the minituber ABA level following chemical inhibition of ABA8′OH activity only partially (but not significantly) delayed minituber dormancy release (Suttle *et al.*, 2012).

In the current study, the hypothesis was tested that ABA is involved in the regulation of grape bud dormancy maintenance/release and that HC exerts its enhancing effect, at least in part, by affecting the bud ABA level.

## Materials and methods

### Plant material

The experiments were conducted with mature buds collected from cordon-trained grapevines (*Vitis vinifera* cv. Early sweet) in a commercial vineyard located in the Jordan Valley. All plants were subjected to the cultural practices commonly used in commercial vineyards.

The grape bud-break response in single-node cuttings appears to be well correlated with bud behaviour on the vine, and it is therefore used as a common and reliable indicator of the dormancy depth of grapevines under forcing conditions (Shulman *et al.*, 1983; Koussa *et al.*, 1994; Lavee and May, 1997; Or and Viloznyi, 1999; Or *et al.*, 2000*a*; Pérez and Lira, 2005; Pérez *et al.*, 2008). Use of this system enables the study of issues related to true dormancy (endodormancy), without the interference of paradormant and ecodormant effects (Lang, 1987). Another advantage is the possibility of working with a large number of buds, providing a proper representation of the dormancy status of a given bud population at a specific point in time during the dormancy cycle. Hence, vines were pruned to three-node spurs, and the detached canes, each carrying nine buds (in positions 4–12), were transferred to the lab. Canes were cut into single-node cuttings, randomly mixed, and groups of 10 cuttings were prepared. Nine groups were used for each treatment.

### Analysis of the effect of ABA on bud break

For ABA treatments, cuttings were both sprayed and immersed in vases with 10 μM or 100 μM ABA (Protone 20 SG™, Valent BioSciences, 20% active S-ABA, Libertyville, USA Israel) solution (150ml in each vase with three groups of cuttings), with addition of 0.02% (v/v) Triton X-100 (Sigma-Aldrich, St Louis, MO, USA). The vases were transferred to a growth chamber and forced at 22 °C under a 14h/10h light/dark regime. After incubation in ABA for 48h or 96h, cuttings were transferred to tap water. The control was treated similarly with 0.02% Triton X-100 solution.

### Induction of dormancy release by chemical and physical stimuli

Following 48h pre-incubation as described above in 100 μM ABA or water, a second treatment was applied, considered as 0h for bud-break monitoring and bud sampling. The treated groups were then returned to water-containing vases and incubated under the above-described forcing conditions for an additional 28 d for bud-break monitoring. Cuttings that were pre-treated with Triton X-100 solution were used for control, HC, AZ, HC, and hypoxia treatments. For control treatments, the cuttings were sprayed again with tap water. For the HC treatment, cuttings were sprayed with 3% (v/v) ‘Dormex’ (SKW, Trostberg, Germany), a commercial formulation containing 49% (w/v) HC. For the AZ treatment, cuttings were sprayed with 2% (w/v) sodium azide (NaN_3_; Sigma-Aldrich). All solutions were formulated in water containing 0.02% Triton X-100 as the wetting agent. For the HS treatment, cuttings were immersed in 50 °C water for 1h. For the hypoxia treatment, cuttings were placed in glass jars containing 150ml of water and equipped with a rubber plug (80 cuttings per 2 litre jar). Jars were flushed with N_2_ to reduce the O_2_ level to 1%. Cuttings were removed from the sealed jars 48h later and transferred to vases as described above.

For the combined ABA–HC, ABA–AZ, ABA–HS, and ABA–hypoxia treatments, cuttings were initially treated with 100 μM ABA for 48h, and then treated with HC, AZ, HS, or hypoxia as described above.

The chemical 2,5-norbornadiene (NBD) binds specifically to ethylene receptors and competes with ethylene for the ethylene-binding sites (Sisler and Serek, 2003). NBD–HC and the relevant HC control were set up in sealed jars under the conditions described above. NBD (Sigma-Aldrich) was placed in a perforated container within each NBD treatment jar (5ml l^–1^) and jars were left sealed for 48h. Cuttings were then removed from the jars, treated with HC or water as described above, and transferred to vases in a growth chamber under the conditions described above.

Bud break was monitored 7, 11, 14, 18, 21, 25, and 28 d after treatment under the forcing conditions described above. Bud break was defined as the stage at which green tissue becomes visible underneath the bud scales. For gene expression and hormone analyses, identical treatments were carried out and buds were sampled at 12, 24, 48, and 96h, frozen in liquid nitrogen, and kept at –80 °C. Buds from jar-based treatments were only sampled at 48h from sealing time.

### Quantitative real-time PCR analyses

Relative transcript levels were measured by quantitative real-time PCR (qRT-PCR) with ABsolute Blue QPCR SYBR Green Low ROX Mix (Thermo Fisher Scientific, Waltham, MA, USA) on a Corbett Rotor-Gene 6000 (Qiagen, Hilden, Germany). Total RNA was extracted from 2g sampled after grinding 20 buds as described previously (Or *et al.*, 2000*a*), and treated with RQ DNase (Promega, Madison, WI, USA) according to the manufacturer’s instructions. First-strand cDNA was synthesized from 2.5 μg of total RNA using Moloney murine leukaemia virus reverse transcriptase (M-MLV RT; Promega) according to the manufacturer’s instructions. VvActin primers, characterized and optimized by Reid *et al.* (2006), were used for normalization.

The 10 μl reaction mixture consisted of 0.1 μM of forward and reverse primers, 5 μl of SYBR-Green (ABsolute Blue qPCR SYBR Green Mixes, Thermo Fisher Scientific), and 4 μl of cDNA diluted 1:32. PCRs were run under the following conditions: 15min at 95 °C and 40 cycles of 15 s at 95 °C, 20 s at 60 °C, and 20 s at 72 °C. No-template controls consisted of all of the above components with the exception of cDNA. For each sample, six independent quantitation analyses comprising three biological repeats with two technical repeats were carried out. All of the primers (Supplementary Table S1 at *JXB* online) were designed by Primer3 software (http://frodo.wi.mit.edu/primer3/).

### Quantitation of endogenous ABA and its catabolites

Triplicate samples of 10 frozen buds for each biological replicate were homogenized in liquid nitrogen, and 0.5g of the homogenized powder was sampled. The sample was extracted with 3ml of 80% methanol containing 1% acetic acid and deuterium-labelled ABA, neophaseic acid (neoPA), phaseic acid (PA), dihydrophaseic acid (DPA), and ABA glucosyl ester (ABA-GE) as internal standards, for 1h at 4 °C. Samples were centrifuged at 3000 *g* for 10min and filtered through an LRC-2 Frits Bond Elut Reservoir (Agilent Technologies, Santa Clara, CA, USA) to remove residual plant materials. The solvent (80% methanol, 1% acetic acid) extraction was repeated for 10min, and samples were centrifuged and filtered as before. The two extracts were combined and evaporated to dryness at 35 °C using a Savant SpeedVac Concentrator (Thermo Fisher Scientific). Dried samples were redissolved in 1ml of 80% acetonitrile, 1% acetic acid. The acetonitrile was removed by evaporation *in vacuo*. ABA and its catabolites were purified and measured as previously described with slight modification (Seo *et al.*, 2011). After purification with a reverse phase column cartridge (Oasis HLB 30mg, 1ml, Waters, Milford, MA, USA), extracts were completely dried for sequential purification with a weak anion exchange column cartridge (BondElut DEA, 100mg, 1ml, Agilent Technologies). Dry residues were dissolved in 1ml of methanol and loaded onto BondElut DEA. Flow through which contains ABA-GE was corrected and then the eluent of methanol containing 1% acetic acid which contains ABA and other catabolites was corrected. The prominent ions for each compound were analysed by a liquid chromatography–tandem mass spectrometry system consisting of an ultra high performance liquid chromatograph (Agilent 1200 UHPLC; Agilent Technologies) and a triple quadrupole mass spectrometer (Agilent 6410; Agilent Technologies) equipped with an ODS column (ZORBAX XDB-C18, 2×50mm, 1.8 μm; Agilent Technologies). Analysis parameters are detailed in Supplementary Table S2 at *JXB* online. The endogenous ABA and catabolite contents were calculated from the peak area ratios of these endogenous compounds to internal standards.

## Results

### Effect of exogenous ABA on dormancy release of grapevine buds

**Fig. 1.**
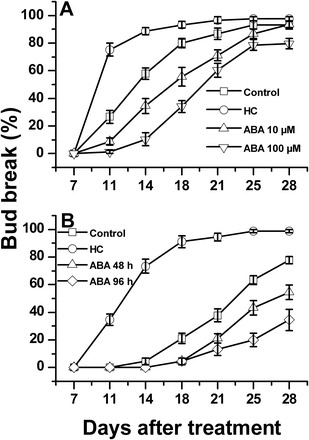
ABA delays bud break in a concentration- and duration-dependent manner. Vines of *Vitis vinifera* cv. Early Sweet from a vineyard at Gilgal, located in the Jordan Valley, were pruned to three-node spurs. The detached canes were cut into single-node cuttings, randomly mixed, and groups of 10 cuttings were prepared. (A) Four treatments were carried out, each with nine groups of 10 cuttings. The bases of the cuttings were immersed in vases containing 10 μM ABA, 100 μM ABA (with 0.02% Triton), or only 0.02% Triton (for control and HC treatments). The vases were placed in a growth chamber and forced at 22 °C under a 14h/10h light/dark regime. After 48h, the solutions were replaced with tap water, and sprayed with 0.02% Triton instead, apart from the HC-treated buds which were sprayed with 3% ‘Dormex’ as detailed in the Materials and methods. The treated groups were forced under the above conditions for another 28 d. Bud break was monitored at 7, 11, 14, 18, 21, 25, and 28 d after spraying. Values are averages of the nine groups in each treatment ±SE. (B) Both ABA treatments were carried out using 100 μM ABA. In the ABA 96h treatment, the cuttings were returned to ABA solution for an additional 48h after spraying. All other details are as in (A).

To test the hypothesis that ABA regulates dormancy release of grapevine buds, the responses of dormant buds to application of ABA, a known inducer of dormancy release (HC), or water were compared. HC application led to the expected enhancement of bud dormancy release relative to the control. ABA, however, had a significant inhibitory effect on dormancy release of the tested bud population (Fig. 1A). Incubation of single-node cuttings with 10 μM ABA for 48h resulted in decreases of 18, 23, 25, and 16% in bud-break percentage relative to the control population at 11, 14, 18, and 21 d after treatment, respectively. Similar treatment with 100 μM ABA resulted in even stronger inhibition, with decreases of 25, 48, 46, and 26% in bud-break percentage relative to the control at the same time points. Incubation in 100 μM ABA for the longer period of 96h resulted in a higher degree of inhibition compared with incubation for 48h under identical conditions (Fig. 1B).

### Effect of ABA on the enhancing effect of various dormancy release stimuli

**Fig. 2.**
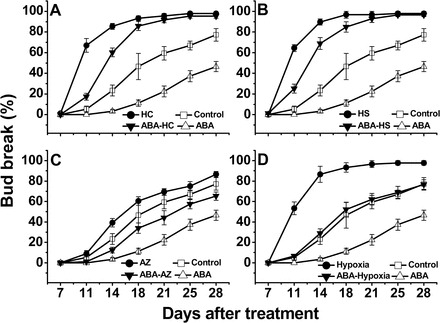
ABA attenuates the enhancing effect of various dormancy release stimuli. (A) In the combined ABA–HC treatment, buds were treated with ABA for 48h prior to HC treatment. All other details are as in Fig. 1. The experimental scheme used in (B), (C), and (D) is identical to that described in (A) apart from the indicated changes. (B) HC treatment was replaced with HS treatment where cuttings were immersed in 50 °C water for 1h as detailed in the Materials and methods. (C) HC treatment was replaced with AZ treatment where cuttings were sprayed with 2% sodium azide and 0.02% Triton. (D) HC treatment was replaced with hypoxia treatment where cuttings were incubated for 48h in sealed jars which were flushed with N_2_ to reduce O_2_ level to 1%.

Based on its inhibitory effect on bud dormancy release, it was suggested that exogenous ABA might also slow the advancing effect of HC on dormancy release of grapevine buds. Compared with the HC treatment, combined treatment with ABA and HC (ABA–HC) attenuated the bud-break rate, producing a ΔBud Break of 50% and 25% at 11 d and 14 d after HC treatment, respectively (Fig. 2A). Interestingly, recovery from such inhibition was evident 21 d after the ABA–HC treatment, compared with the HC-treated buds, whereas no recovery was evident for ABA-treated buds compared with the control. It should be noted that the ABA–HC treatment produced higher levels of bud break than the controls at all analysed time points.

Similar analyses were carried out to test the effect of ABA on the effect of other known stimuli of dormancy release. Incubation in 100 μM ABA for 48h prior to treatment with AZ, application of HS, or incubation under hypoxia for 48h attenuated bud break compared with the respective treatments with the relevant inducer of dormancy release without ABA (Fig. 2B–D). Similar to ABA–HC, recovery was evident for the ABA–HS-treated buds. However, the ABA–AZ and ABA–hypoxia buds failed to reach the bud-break percentage of the AZ or hypoxia treatments during the analysed period. Unlike the ABA–HC- and ABA–HS-treated bud populations, the ABA–AZ and ABA–hypoxia populations did not present higher levels of bud break than their respective controls at any analysed time point.

### Effect of HC on expression of central components of ABA metabolism in grape buds

In light of the described findings, it was speculated that ABA might be involved in repression of primordial growth, and that stimuli of dormancy release, such as HC, may be involved in diminishing its repression potential via modification of ABA metabolism. To test this assumption, comparative transcript profiling of central regulators of ABA synthesis and degradation was carried out.

Previous bioinformatics analyses identified three putative grape homologues of *NCED* (*VvNCED*) and eight homologues of the *Arabidopsi* ***s*** *ABA8′OH* gene (*VvA8H-CYP707A*), encoding rate-limiting enzymes in ABA biosynthesis and catabolism, respectively (Young *et al.*, 2012). In the current study, a single homologue of *XERICO*, termed *VvXERICO*, was identified (Supplementary Fig. S2 at *JXB* online). In mature grape buds, expression of all three homologues of *NCED* (hereafter referred to as *VvNCED1*, *VvNCED2*, and *VvNCED3*) was detected, but the levels of the latter two were very low compared with that of *VvNCED1*. Expression of *VvXERICO*, *VvA8H-CYP707A1*, and *VvA8H* *-CYP707A4* was also recorded; expression of *VvA8H-CYP707A2* was also detected, but only at very low levels.

**Fig. 3.**
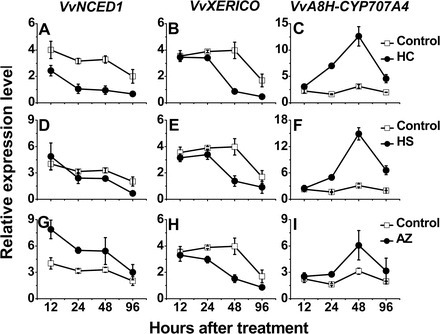
Dormancy release stimuli modulate transcription of regulators of ABA metabolism. Total RNA was extracted from control, HC-, HS-, and azide-treated buds sampled 12, 24, 48, and 96h after treatment. Relative expression levels of *VvNCED1*, *VvXERICO*, and *VvA8H-CYP707A4* were determined by qRT-PCR as described in the Materials and methods and normalized against *VvActin*. The values represent the mean ±SE of three biological repeats, each with two technical repeats. Relative expression levels are presented for HC versus control buds (A–C), HS versus control buds (D–F), and AZ versus control buds (G–I) sampled 12, 24, 48, and 96h after treatment.

Analyses of the effect of HC on the transcript levels of *VvXERICO* and the bud-expressed members of the *VvNCED* and *VvA8H-CYP707A* gene families were carried out using qRT-PCR. In agreement with a previous microarray analysis (Ophir *et al.*, 2009), HC treatment seemed to down-regulate the expression of *VvNCED1* and *VvXERICO* significantly, with a maximum difference at 48h (Fig. 3A, B). In contrast, HC led to a significant increase in the transcript level of *VvA8H-CYP707A4*, which peaked at 48h (Fig. 3C). A similar trend, but with less pronounced differences, was recorded for transcript levels of *VvNCED2* (Supplementary Fig. S3A at *JXB* online), *VvNCED3* (Supplementary Fig. S3B), and *VvA8H-CYP707A1* (Supplementary Fig. S3C). Parallel profiling of *VvNCED1* (Fig. 3D), *VvXERICO* (Fig. 3E), and *VvA8H-CYP707A4* (Fig. 3F) in HS-treated buds presented very similar results relative to their controls. In AZ-treated buds, however, only the profiles of *VvXERICO* (Fig. 3H) and *VvA8H-CYP707A4* (Fig. 3I) agreed with the patterns presented for HC and HS, whereas transcript levels of *VvNCED1* were higher in the AZ-treated buds than in the control (Fig. 3G). At 12h, no difference was detected apart from down-regulation of *VvNCED1* transcript levels by HC and up-regulation by AZ.

### Effect of HC on expression of central components of ABA signalling in grape buds

**Fig. 4.**
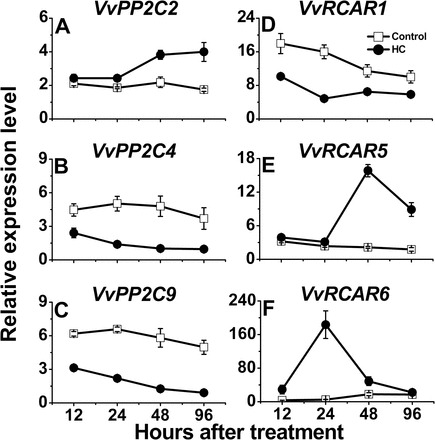
HC modulates transcription of central components of ABA signalling. Relative expression levels of *VvPP2C2* (A), *VvPP2C4* (B), *VvPP2C9* (C), *VvRCAR1* (D), *VvRCAR5* (E), and *VvRCAR6* (F) are presented in HC and control buds sampled at 12, 24, 48, and 96h after treatment. All other details are as described in Fig. 3.

The gene families of the central players in ABA signalling in *V. vinifera* have been recently identified and characterized (Boneh *et al*., 2012*a*, *b*). In the current study, analyses are presented of the effect of HC on the transcript levels of members of the ABA receptors, PP2C and ABA-responsive element/ABA binding factor (AREB/ABF) gene families in the buds. Overall, the data suggest that HC triggered reprogramming of the expression of these ABA signalling components which are known to be regulated at the transcriptional level (Kuhn *et al.*, 2006; Santiago *et al.*, 2009; Yoshida *et al.*, 2014). While levels of *VvPP2C4* (Fig. 4B), *VvPP2C9* (Fig. 4C), and *VvRCAR1* (Fig. 4D) transcripts were significantly reduced in HC-treated buds, levels of *VvPP2C2* (Fig. 4A), *VvRCAR5* (Fig. 4E), and *VvRCAR6* (Fig. 4F) transcripts were markedly induced.

S**ignificant but smaller changes were** recorded in the transcript levels of *VvRCAR2* (Supplementary Fig. S4A at *JXB* online) and *VvRCAR7* (Supplementary Fig. S4D), which were up-regulated in response to HC. No clear difference was observed in the level of *VvRCAR3* (Supplementary Fig. S4B **) and** *VvRCAR4* (Supplementary Fig. S4C).

**Fig. 5.**
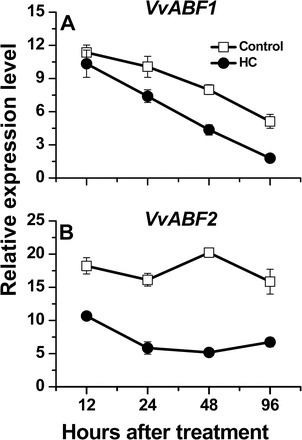
HC modulates expression of the central ABA response mediators *VvABF* genes. Relative expression levels of *VvABF1* (A) and *VvABF2* (B) are presented in HC and control buds sampled at 12, 24, 48, and 96h after treatment. All other details are as described in Fig. 3.

Analysis of the effect of HC on the transcript levels of *AREB/ABF* genes identified in grapevine (Boneh *et al.*, 2012*a*) indicated that both *VvABF1* and *VvABF2* are significantly down-regulated in response to HC (Fig. 5).

### Effect of HC treatment on endogenous ABA and ABA catabolite content of grapevine buds

**Fig. 6.**
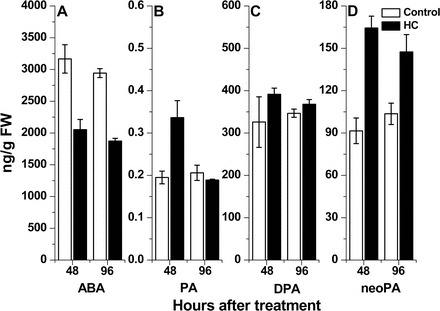
Effect of HC on ABA and ABA catabolite contents in grapevine buds. ABA (A), PA (B), DPA (C), and neoPA (D) levels were determined in HC-treated and control buds sampled 48h and 96h after treatment. The homogenized samples (0.5g) were used for hormone extraction as detailed in the Materials and methods, and ^2^H-labelled ABA, PA, DPA, and neoPA were spiked as internal standards. Levels of ABA and its catabolites were analysed by liquid chromatography–tandem mass spectrometry. The levels of the analysed molecules were calculated from the peak area ratios of the endogenous molecule to the relevant internal standard. Values represent means ±SE of three biological repeats (10–12 buds per repeat).

The levels of endogenous ABA and its catabolites neoPA, PA, and DPA were determined in HC-treated and control buds sampled at 48h and 96h after treatment (Fig. 6). Compared with controls, HC treatment resulted in a 35% decrease in endogenous ABA level (Fig. 6A). On the other hand, levels of neoPA were 1.8- and 1.4-fold higher in the HC-treated buds at 48h and 96h, respectively, compared with the control (Fig. 6D). PA and DPA levels were higher in HC-treated buds at 48h (1.72- and 1.2-fold, respectively), but at 96h their levels decreased and were similar to those of the control buds, which presented rather stable levels throughout the analysed period (Fig. 6B, C). It should be noted that levels of PA in the buds were 1000-fold lower than those of neoPA and DPA. Levels of ABA-GE were similar in HC-treated and control buds at the analysed time points (data not shown).

**Fig. 7.**
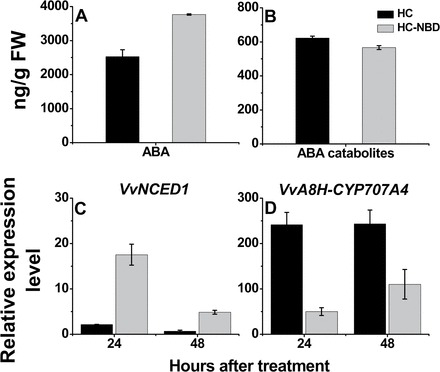
An inhibitor of ethylene signalling attenuates the enhancing effect of HC on ABA down-regulation. Levels of ABA (A) and ABA catabolites (B) were determined as described in Fig. 6 in HC- and NBD–HC-treated buds sampled 48h after treatment. Levels of *VvNCED1* (C) and *VvA8H-CYP707A4* (D) transcript were determined in the same bud samples as described in Fig. 3.

Interestingly, exposure of HC-treated buds to the ethylene signalling inhibitor NBD for 48h led to a 1.49-fold increase in ABA level (Fig. 7A), and a 1.1-fold decrease in ABA catabolites (Fig. 7B) compared with HC-treated buds. Transcription profiling revealed that, in accordance with the attenuation in ABA degradation exerted by NBD in HC-treated buds, the treatment also attenuated the HC-induced down-regulation of *VvNCED1* (Fig. 7C) and up-regulation of *VvA8H-CYP707A4* (Fig. 7D).

### Profiling of *VvNCED1* and *VvA8H-CYP707A4* transcript levels during the dormancy cycle

**Fig. 8.**
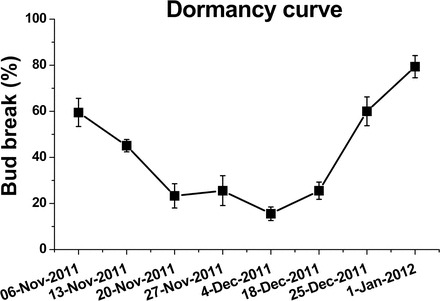
Changes in dormancy status of the bud population throughout the dormancy cycle. Canes from the vineyard under study were sampled weekly during the dormancy cycle. Single-node cuttings were prepared and bud break was monitored as described in Fig. 1. Bud-break percentages at 21 d are presented to describe the seasonal changes in dormancy status of the bud population in the vineyard. Values are averages of nine groups of replications, consisting of 10 buds each ±SE.

To assess the potential involvement of ABA level and metabolism in the execution of natural dormancy, the dormancy status of buds was assessed from the beginning of November to the beginning of January (Fig. 8). An ~40% decrease in bud-break percentage from the beginning of November to 20 November might pinpoint this as the dormancy induction period. The period of dormancy maintenance, with bud-break percentages of 15–25%, lasts through the last third of November to 18 December, with maximal dormancy depth occurring in the middle of that period (4–11 December). During the last third of December, repression is alleviated, as reflected by the increasing percentage of bud break.

**Fig. 9.**
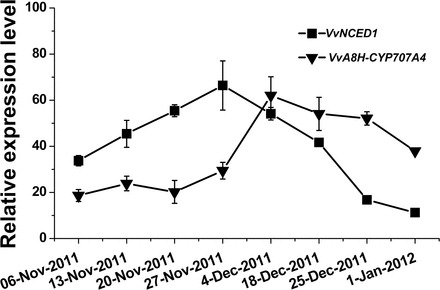
Expression profile of *VvNCED1* and *VvA8H-CYP707A4* throughout the dormancy cycle. Buds were sampled from canes that were harvested weekly during the dormancy cycle as described in Fig. 8. Levels of *VvNCED1* and *VvA8H-CYP707A4* transcripts were determined as described in Fig. 3.

Levels of *VvNCED1* and *VvA8H-CYP707A4* transcripts were monitored in buds sampled throughout this natural dormancy cycle (Fig. 9). The level of *VvNCED1* gradually increased and peaked in the last third of November (27 November), when bud-break percentage was ~25%. At the beginning of December (4 December), when the bud population reached its maximal dormancy (15% bud break for the analysed season), the level of *VvNCED1* transcript started dropping, reaching its lowest level during maximum dormancy release in January. Concomitantly, the transcript level of *VvA8H-CYP707A4*, which was constantly low until the end of November, sharply increased and remained high during the period of dormancy release.

### Levels of ABA, neoPA, PA, and DPA in grape buds during the dormancy cycle

**Fig. 10.**
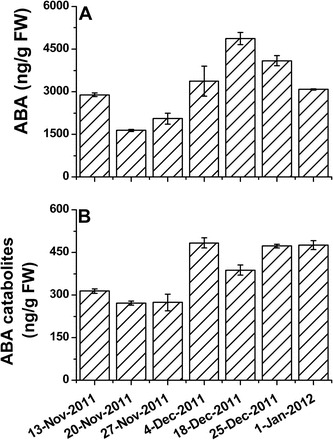
Changes in the contents of ABA and its catabolites throughout the dormancy cycle. The contents of ABA (A) and ABA catabolites (B) were determined as described in Fig. 6 in buds sampled at seven time points throughout the dormancy cycle. Sampling and dormancy status are described in Fig. 8.

Levels of ABA and its catabolites were determined in grape buds sampled throughout the natural dormancy cycle, from mid-November to the beginning of January. ABA levels increased ~3-fold from 20 November to 18 December, and then decreased to 60% of maximum in the following 2 weeks (Fig. 10A) in parallel with an increase in bud-break percentage from 25% to 80% (Fig. 8).

The data presented in Fig. 10B suggest that the level of ABA catabolites was significantly increased between 27 November and 4 December, concomitant with the sharp increase in *VvA8H-CYP707A4* transcript level (Fig. 9), and remained consistently high until the end of the analysed period.

### Differential effect of ABA treatment on bud dormancy release during the natural dormancy cycle

**Fig. 11.**
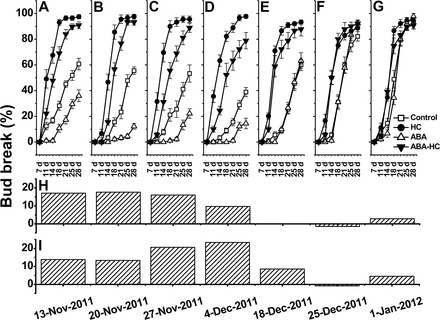
Differential effects of ABA treatment on bud dormancy release during the natural dormancy cycle. Single-node cuttings were prepared from canes that were harvested at seven sampling dates throughout the dormancy cycle as described in Fig. 8. Cuttings were grouped as described in Fig. 1 and used to analyse the effect of ABA on dormancy release of control and HC-treated buds as described in Fig. 2A. In parallel with the actual bud-break data (A–G), calculated values are presented as the difference in bud-break percentages between control and ABA-treated buds (H), and between HC- and ABA–HC-treated buds (I). These values represent the mean of differences for seven monitoring time points (7, 11, 14, 18, 21, 2, and 28 d) for each sampling date.

To advance understanding of the potential role of ABA in regulating the dormancy cycle, the effect of exogenous ABA on natural and HC-stimulated dormancy release was analysed independently at several time points during the dormancy cycle, using the single-node cutting experimental system. In parallel with the actual bud-break data (Fig. 11A–G), the differences in bud-break percentage between pairs of relevant treatments are presented in Fig. 11H and I. As expected, the data indicated that compared with controls, HC enhancement of dormancy release increases as dormancy deepens, and its effect decreases toward natural dormancy release (see Fig. 8 for the seasonal dormancy curve, where deepest dormancy in the given season was represented by 15% bud break at 21 d). The data also indicated that compared with controls, the inhibitory effect of exogenous ABA decreases as dormancy deepens, and inhibition is not evident during natural dormancy release.

While exposure to exogenous ABA reduced the advancing effect of HC, the extent of this reduction increased until the onset of maximal dormancy, and then gradually decreased during the phase of dormancy maintenance and the initial stages of dormancy release. During the natural dormancy release phase, exogenous ABA lost its inhibitory effect on dormancy release of HC-treated buds. Earlier, it was seen that HC enables recovery from the inhibitory effect of ABA (Fig. 2A). The data presented in Fig. 11 suggest that the timing of the recovery from ABA repression is delayed as dormancy progresses.

## Discussion

### Exogenous ABA delays bud dormancy release

The hypothesis that ABA is involved in regulating the maintenance of grape bud dormancy and that HC exerts it enhancing effect, at least in part, by affecting bud ABA level was tested. In agreement with this hypothesis, the presented results suggest that exogenous ABA delays bud break of dormant buds (Fig. 1). The degree of inhibition seems to be dependent on ABA concentration, and on duration of incubation, supporting causal relationships between inhibition and ABA. These results are in agreement with the negative effects of exogenous ABA on seed germination (Rubio *et al.*, 2009; Santiago *et al.*, 2009; Ye *et al.*, 2011), and on bud break in willow (Barros and Neill, 1989), apple (Dutcher and Powell, 1972), pear (Tamura *et al.*, 2002), kiwi (Lionakis and Schwabe, 1984), and sour cherry (Mielke and Dennis, 1978).

Since ABA application to buds that are no longer dormant did not delay emergence of the primordial shoot (Fig. 11F, G), the reported inhibitory effect cannot be considered a non-specific and wide-ranging suppressive effect on bud primordial growth activity. Alternatively, such inhibition may be viewed as a component of a unique and complex mechanism that controls meristem activity during a specific developmental stage of the grapevine life cycle. In agreement with this, the degree of inhibition exerted by exogenous ABA seems to be affected by the dormancy status of the analysed bud population, as shown by ABA’s decreased ability to inhibit bud burst as buds reach deep dormancy, and then progress toward natural dormancy release. This further supports the assumption that the inhibitory effect of ABA is part of a wider regulatory network that operates during the dormancy cycle.

### ABA limits the enhancing effect of HC and other dormancy release stimuli

The ability of HC and other artificial stimuli to enhance dormancy release of grape buds has been previously documented (Ophir *et al.*, 2009 and references within) and was confirmed in the current study. The delay exerted by exogenous ABA on the advancing effect of HC, HS, AZ, and hypoxia (Fig. 2) supports the assumption that ABA has a critical role in maintaining grape bud dormancy, and suggests that it inhibits the cascade of biochemical changes activated by the artificial dormancy release stimuli that lead to dormancy release. In support of this, the stimulatory effect of H_2_O_2_ on seed dormancy release is negatively affected by exogenous ABA (Sarath *et al.*, 2007). The recovery of ABA–HC- and ABA–HS-treated buds from this inhibitory effect 18 d or more post-treatment, in contrast to the behaviour of ABA-treated buds, suggests that HC- and HS-treated buds recruit the ability to deal with increased levels of ABA, possibly by affecting ABA metabolism and/or sensing.

### HC affects ABA metabolism

HC induced a reduction in the transcript levels of *VvXERICO* and two *VvNCED* genes, as well as a parallel induction of two *VvA8H-CYP707A* homologues, suggesting that it exerts at least part of its enhancing effect through modification of ABA metabolism, resulting in a reduction in the total level of ABA. The mode of regulation suggested by the changes recorded in transcript levels is in agreement with the final outcome in metabolite level, as reflected by the decrease in bud endogenous ABA and parallel increase in ABA degradation products in HC-treated buds (Fig. 6). Taken together, these results support the hypothesis that HC treatment leads to a decrease in endogenous ABA level by promoting ABA degradation, inhibiting ABA synthesis, or both. This hypothesis is supported by the effects of dormancy release stimuli on the ABA degradation machinery in other systems. The stimulation of arabidopsis seed dormancy release by H_2_O_2_, nitrate, and nitric oxide (NO) is mediated largely by ABA8′OH, which catalyses the degradation of endogenous ABA (Liu and Zhang, 2009; Matakiadis *et al.*, 2009; Liu *et al.*, 2010). Bromoethane, which induces sprouting of dormant potato buds, led to a significant decrease in meristem ABA content and increase in ABA catabolism, which occurred predominantly via oxidation catalysed by ABA8′OH. The increased level of *StCYP707A* transcript in the meristem in response to bromoethane is consistent with the latter’s effect on ABA level (Destefano-Beltrán *et al.*, 2006*a*). Changes in ABA content in whole potato tubers were also recorded following enhancement of dormancy release by synthetic cytokinin or heat stress (Ji and Wang, 1988; van Den Berd *et al.*, 1991). In agreement with this, increased exposure to controlled chilling, a natural stimulus of dormancy release, led to a decrease in ABA levels in pear vegetative buds (Tamura *et al.*, 2002).

### The inhibitory effect of ABA is sensitive to seasonal changes

As the season progressed, a decrease was recorded in the degree of inhibition exerted by exogenous ABA on dormancy release of both control and HC-treated buds (Fig. 11). The results are supported by the periodicity of the response to ABA in lateral buds of willow (Barros and Neill, 1989). Additional support stems from the inhibitory effect of ABA on bud break of dormant pear buds exposed to 200–500 chilling hours, and its inability to affect bud burst of similar buds that present shallow dormancy after exposure to 800–1000 chilling hours (Tamura *et al.*, 2002). This decreased response, which supports the assumption that ABA’s effect is dependent on developmental stage, may be explained by each of the following scenarios, or some combination of them: (i) an increase in total ABA level beyond that required for maximal repression, as a result of an increased endogenous ABA level; (ii) an increase in ABA degradation capacity which facilitates more efficient removal of the added exogenous ABA; and (iii) developmental phase transition, which leads to the establishment of a new regulatory network, where ABA is no longer a regulator of primordial growth activity. Transition from one scenario to another is expected and assumed to be possible during the dormancy cycle. A gradual increase in *VvNCED1* transcript and endogenous ABA levels up to a maximum at the stage of dormancy maintenance (Figs 8, 9) support the first scenario. The correlation between the degree of inhibition by exogenous ABA (reflected by ΔCon–ABA, presented in Fig. 11H) and the endogenous ABA levels also suggests that the effect of exogenous ABA decreases with a rise in endogenous ABA. A sharp increase in the levels of *VvA8H-CYP707A4* transcript and ABA degradation products in the heart of the dormancy maintenance period supports the second scenario. The complete inability of ABA, as well as HC, to affect bud burst toward the phase of natural dormancy release (Fig. 11G) may favour the third scenario. The parallel decrease in the level of *VvNCED1* transcript and increase in the levels of both *VvA8H-CYP707A4* transcript and ABA degradation products from 27 November to 4 December may serve as an initial indication of the existence of a defined developmental window in the bud dormancy cycle when ABA can play a regulatory role in dormancy maintenance. In line with this, it is suggested that the maximal difference between HC and ABA–HC treatments (ΔHC–ABAHC, Fig. 11I) reflects both the deepest natural dormancy and maximal enhancing effect of HC, before the buds become sensitive and HC damage masks potential bud-break ability.

Unfavourable light or temperature conditions have been shown to prevent germination by co-ordinated regulation of *NCED* and *CYP707A* gene expression in several species (Seo *et al.*, 2006; Gubler *et al.*, 2008; Toh *et al.*, 2008; Leymarie *et al.*, 2009; Argyris *et al.*, 2011). Interestingly, a wider group of key *Arabidopsis* genes, which are involved in the regulation of ABA metabolism and signalling, has recently been shown to be highly sensitive to slow seasonal changes, and regulation of their expression in seeds in response to soil temperature results in continual and dramatic adjustments to the dormancy depth within the soil seed bank. Among these are *NCED6*, *SnrK2.1*, *SnrK2.4*, and *ABI3*, which are up-regulated when temperatures are low and lead to deep dormancy. The transition to shallow dormancy is linked to ABA catabolism and repression of ABA signalling, as evidenced by the increased expression of *CYP707A2* and *ABI2* in response to high soil temperature and dormancy release (Footitt *et al.*, 2011). Co-ordinated regulation of the levels of both ABA and ABA metabolism regulators during the dormancy cycle has also been shown in a few bud studies. ABA levels in apical poplar buds increased significantly after 3–4 weeks of short days, which induce dormancy (Rohde *et al.*, 2002), in parallel with significant up-regulation of genes encoding NCED and other enzymes catalysing ABA biosynthesis (Ruttink *et al.*, 2007). Significant seasonal changes in ABA content, which negatively correlated with bud-burst ability, were also recorded in the apical buds of silver birch (Rinne *et al.*, 1994*b*). In potato tuber meristems, ABA content rose significantly as the natural dormancy cycle progressed, and then decreased steadily. These changes were positively correlated with changes in the expression of *StNCED2*, whereas expression of *StCYP707A1* was up-regulated when the ABA level started to decrease (Destefano-Beltrán *et al.*, 2006*b*).

It should be noted that the changes in ABA level lagged somewhat behind the changes in *VvNCED1* and *VvA8H-CYP707A4* transcript levels, as well as the levels of ABA catabolites. The technical failure to determine the levels of ABA and its degradation products on 11 December may have prevented the detection of a potentially higher and earlier ABA peak. Another option is that VvNCED1 protein level or activity is not completely mirrored by the level of its transcript, allowing an extended period of ABA synthesis, and temporarily masking the effect of increased degradation ability. Along the same lines, changes in ABA levels in potato tuber meristems were reported to lag behind increased expression of *StCYP707A*, and it was speculated that ABA8′OH activity might also be regulated post-transcriptionally (Destefano-Beltrán *et al.*, 2006*a*). It is clear, however, that ABA quantity rises to a maximal level at the stage of dormancy maintenance (from 20 November to 18 December) and gradually decreases in parallel with increasing natural bud-break ability, in agreement with the initial hypothesis. Based on the data, it can be speculated that upon induction of dormancy by as yet unidentified environmentally regulated factors, preparation for ABA production starts at the level of transcription and actual accumulation of ABA starts later, serving as a master regulator of dormancy maintenance. Future production of NCED antibodies and/or analysis of NCED activity will enable this assumption to be tested.

Despite the delayed bud break with the combined treatments of ABA–HC and ABA–HS compared with HC, bud break occurred at higher levels compared with controls at all time points. This behaviour is in agreement with the suggested amplification of ABA degradation ability by HC and HS treatments, which may allow the buds to process higher levels of ABA (both endogenous and exogenous) with better efficiency than control buds. It is speculated that by increasing the level of exogenous ABA beyond the processing ability of the HC-treated buds, its inhibitory effect will be intensified. Experiments with ABA concentrations >100 μM were not conducted, but delayed recovery following ABA–HC treatment with extended incubation in ABA (6 d) supports this assumption (data not shown).

It is interesting to note that the inhibitory effect of ABA on enhancement of dormancy release by AZ and hypoxia was greater than that recorded for HC or HS, as reflected by (i) the inability of ABA–AZ- and ABA–hypoxia-treated buds to present bud-break levels that are higher than that of the control; and (ii) the absence of recovery of the AZ- and hypoxia-treated buds to comparable levels, which was evident in the ABA–HC and ABA–HS treatments. In the case of AZ, this may stem from a degree of phytotoxicity resulting from vigorous stress. This scenario agrees with the limited enhancement of dormancy release by 2% AZ (Fig. 2C), versus the better enhancement recorded at lower concentrations (E. Or *et al*., unpublished results). It is speculated that such harsh stress may increase ABA levels (as reflected by an increased *VvNCED1* transcript level instead of the expected decrease), and thus delay removal of inhibition. Currently, there are no data that coincide with the potentially lower ability of the hypoxia treatment to enhance ABA degradation relative to HC and HS.

### Potential involvement of ABA signalling components in the regulation of dormancy release

Although the reported results strongly support regulation at the level of ABA metabolism, potential changes in ABA signalling are also possible. Thus, several candidates from the gene families of central players in the ABA signalling machinery were selected for transcript profiling. The selection was based on (i) previous studies suggesting that ABA receptor, PP2C, and ABRE genes are regulated at the transcriptional level (Kuhn *et al.*, 2006; Santiago *et al.*, 2009; Raghavendra *et al.*, 2010; Szostkiewicz *et al.*, 2010; Footitt *et al.*, 2011; Yoshida *et al.*, 2014); (ii) previous identification of family members that are regulated by ABA and present potential protein–protein interactions with their targets in the ABA signalling cascade in grapevine (Boneh *et al*., 2012*a*, *b*); and (iii) validation of the expression of the selected candidates in grapevine buds. It has been shown that under conditions that increase ABA levels, the transcript levels of the ABA receptors are down-regulated and the level of *PP2C* transcript is increased due to feedback regulation (Raghavendra *et al.*, 2010; Szostkiewicz *et al.*, 2010). The HC-induced reduction of *VvNCED1* transcript on the one hand, and increase in level of *VvA8H-CYP707A4* transcript, DPA, and neoPA on the other, were linked to the decrease in ABA level. In light of this, induction of all of the receptors except *VvRCAR1*, and down-regulation of *VvPP2C4* and *VvPP2C9* by HC was expected. These changes might reflect a feedback response to a decreased ABA level, and serve as additional validation for ABA-related changes in response to HC. Alternatively, they may be regulated by an as yet unknown master regulator of dormancy status and play a primary role in modifying the cell’s sensitivity to ABA. Notably, while changes in ABA levels were in line with dormancy depth, these changes could not fully explain the seasonal dormancy cycling in *Arabidopsis* seeds, and it was suggested that factors that regulate ABA signalling and sensitivity, such as DOG1 and MFT, must play important roles in seasonal cycling (Footitt *et al.*, 2011). In terminal buds of poplar, ABA biosynthesis and part of the signal transduction pathway are activated concomitantly with the transition of the apex to a closed bud structure, before termination of meristematic activity (Ruttink *et al.*, 2007). Interestingly, the suggestion was raised that ABA signalling might be involved in the regulation of poplar bud sensitivity to the sugar signals that regulate the dormancy cycle (Rohde *et al.*, 2002). The experimental data presented in the current work may serve as an initial indication for possible involvement of ABA signalling in the regulation of grape bud dormancy. However, it should be clearly stated that further research is required to support this assumption fully.

### Removal of ABA occurs downstream of the development of respiratory stress and ethylene signals

The fact that ABA inhibited dormancy release of buds subjected to anaerobic conditions is in agreement with the working model, which suggests that removal of ABA is downstream of the development of respiratory stress. Levels of ABA were not measured in buds subjected to anaerobic conditions, but the increased level of *A8H-CYP707A4* transcript in these buds compared with controls (data not shown) further supports the model. Based on the present model, it is also assumed that ethylene signalling is required to induce ABA degradation. The higher level of ABA and lower levels of ABA catabolites in HC–NBD-treated buds compared with HC-treated buds, coupled with the fact that dormancy release is inhibited by NBD (Ophir *et al.*, 2009), support this assumption. An antagonistic interaction between ethylene and ABA during seed germination has been shown in various species and was recently reviewed by Arc *et al.* (2013). In agreement with the present findings, seeds of ethylene-insensitive *Arabidopsis* mutants *etr1* and *ein2* exhibit a higher ABA content than the wild type and slower germination (Beaudoin *et al.*, 2000; Ghassemian *et al.*, 2000; Chiwocha *et al.*, 2005; Wang *et al.*, 2007). Moreover, *NCED3* up-regulation and *CYP707A2* down-regulation were recorded in *ein2* and *etr1-1* mutants (Cheng *et al.*, 2009). Unlike the suggested antagonistic effect during dormancy release, a synergistic effect was suggested during preparation for bud dormancy, based on data reported for birch. Transgenic ethylene-insensitive birch trees exposed to short days did not accumulate ABA in apical buds, and formation of terminal buds was abolished as well, in contrast to the typical behaviour of birch exposed to such conditions (Ruonala *et al.*, 2006).

### Variability in the response of gene family members to dormancy release stimuli

The present results suggested that only some of the genes in the gene families under study are regulated in the buds during the dormancy cycle, and in response to dormancy release stimuli. Similar scenarios have been described previously in seeds and buds. In barley, transcript levels of *HvNCED1*, but not *HvNCED2*, vary during grain development, and modulate ABA accumulation at late maturation stages and in response to changes in environmental conditions (Chono *et al.*, 2006). In *Arabidopsis*, *NCED6*, *NCED9*, and *CYP707A2* seem to be major players in the regulation of seed dormancy, and opposite profiles were recorded for the receptors *PYR1* and *PYL7* (Lefebvre *et al.*, 2006; Footitt *et al.*, 2011; Frey *et al.*, 2012). In potato tuber meristems, changes in ABA content during progression of the natural dormancy cycle and in response to bromoethane closely mirrored the expression of *StNCED2*, but not that of *StNCED1*. Similarly, decreases in ABA content correlated mainly with *StCYP707A2*, one of three members of the ABA 8′-hydroxylase gene family (Destefano-Beltrán *et al.*, 2006*a*, *b*).

### Final remarks

To summarize, the following scenario is suggested: at early stages of the dormancy cycle, endogenous ABA levels are below the threshold needed to inhibit bud break, and thus a supply of exogenous ABA may have a significant additive effect on the dormancy level. Later, the level of endogenous ABA rises above that threshold, and therefore addition of exogenous ABA gradually loses it additive inhibitory effect. Once ABA degradation abilities are acquired (and levels of synthesis decrease), both endogenous and exogenous ABA are efficiently metabolized, promoting similar dormancy release in both ABA-treated and control buds. In the presence of HC, the degree of recovery from exogenous ABA inhibition, which is facilitated by HC-induced ABA degradation, depends on the endogenous ABA metabolism. Recovery slows down when endogenous ABA levels rise, due to the need for the limited ABA degradation capacity, induced by HC, to handle higher levels of ABA (from combined endogenous and exogenous sources). Later, when endogenous ABA synthesis decreases and ABA degradation naturally increases, the ability to recover is improved, until it becomes irrelevant due to regeneration of full bud-break capacity.

## Supplementary data

Supplementary data are available at *JXB* online.

Figure S1. Reprograming during artificially induced dormancy release: model of current working hypothesis.

Figure S2. Identification of *VvXERICO*.

Figure S3. Transcription modulation of additional bud-expressed *VvNCED* and *VvA8H-CYP707A* genes by hydrogen cyanamide (HC).

Figure S4. Transcription modulation of additional bud-expressed *VvRCAR* genes by hydrogen cyanamide (HC).

Table S1. Primers used for gene expression analyses by qRT-PCR.

Table S2. Parameters for LC-ESI-MS/MS analysis.

Supplementary Data
