# Supplementary material for: Abscisic acid (ABA) regulates grape bud dormancy, and dormancy release stimuli may act through modification of ABA metabolism
Source: J Exp Bot. 2015 Jan 5;66(5):1527–42. doi: 10.1093/jxb/eru519 (PMC4339608; doi:10.1093/jxb/eru519)
Supplement: Supplementary Data [file supp_66_5_1527__index.html]

Abscisic acid (ABA) regulates grape bud dormancy, and dormancy release stimuli may act through modification of ABA metabolism — Abscisic acid (ABA) regulates grape bud dormancy, and dormancy release stimuli may act through modification of ABA metabolism — Supplementary Data 

# Abscisic acid (ABA) regulates grape bud dormancy, and dormancy release stimuli may act through modification of ABA metabolism

## Supplementary Data

Data files

**Files in this Data Supplement:**

- Supplementary Data - Supplementary Data
