# Supplementary material for: Abscisic acid (ABA) regulates grape bud dormancy, and dormancy release stimuli may act through modification of ABA metabolism
Source: J Exp Bot. 2015 Jan 5;66(5):1527–42. doi: 10.1093/jxb/eru519 (PMC4339608; doi:10.1093/jxb/eru519)
Supplement: Supplementary Data [file supp_eru519_jexbot136093_file001.pdf]

ABA regulates grape bud dormancy and dormancy-release stimuli may act through modification of ABA metabolism. *Chuanlin Zheng, Tamar Halaly, Atiako Kwame Acheampong, Yumiko Takebayashi, Jikumaru Yusuke, Yuji Kamiya, Etti Or*

SUPPLEMENTARY DATA

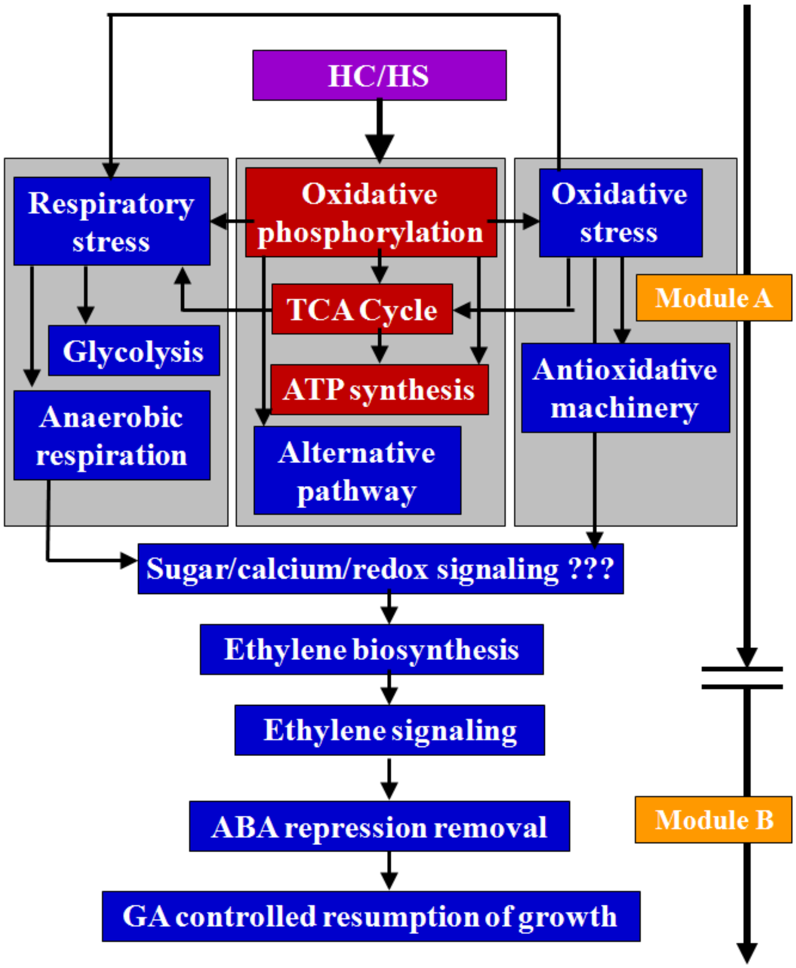

Supplementary Figure S1: Reprograming during artificially induced dormancy release: model of current working hypothesis. According to the model, perturbation of cytochrome-pathway activity within the mitochondria leads to respiratory and oxidative stress, expressed as an increase in the levels of reactive oxygen species, decreased activity of the tricarboxylic acid cycle, and decreased production of ATP. To address this energy crisis, the alternative oxidase pathway, glycolysis, pyruvate metabolism and anaerobic respiration are induced, in an order that has yet to be defined. In parallel, the cellular antioxidant machinery and related pathways are upregulated to cope with the oxidative burst. Changes in redox, sugar, and Ca^{2+} metabolism, resulting from the above reprogramming under conditions that mimic hypoxia, may be responsible for induction of ethylene biosynthesis. Such changes may then affect the interplay between ethylene, abscisic acid (ABA) and gibberellin (GA), such that ABA repression on meristem activity is removed and changes in cell-wall metabolism are induced, leading to cell enlargement. Module A of the cascade regulates induction of ethylene synthesis. Module B is triggered by the ethylene signal, which activates dormancy release via removal of ABA's repression of meristem activity. Blue background: upregulation, Red background: downregulation. HC, hydrogen cyanamide; HS, heat shock.

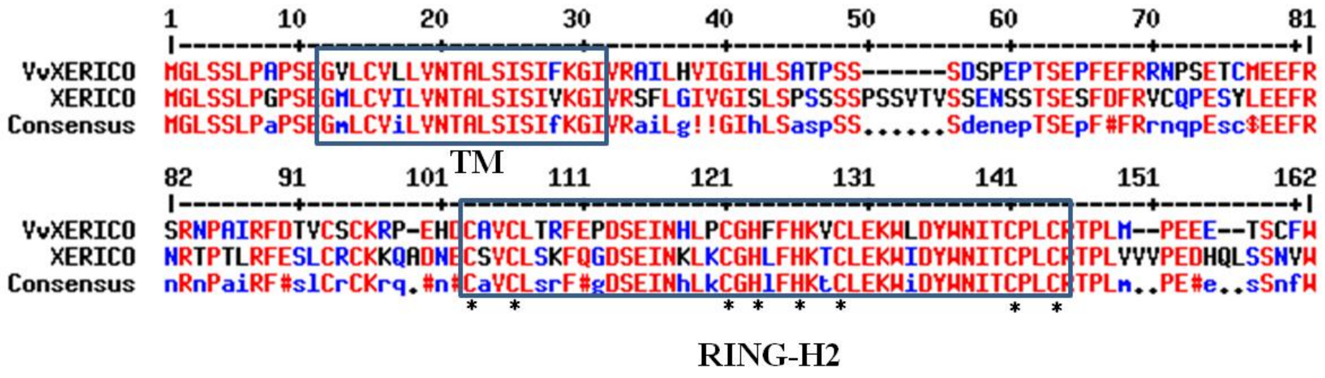

Supplementary Figure S2: Identification of *VvXERICO*. Arabidopsis *XERICO* is considered as a positive regulator of ABA level (Ko et al., 2006). The intronless arabidopsis gene encodes a 162-amino acid protein. Using the arabidopsis amino acid sequence, one putative grapevine homolog was identified by blasting of the NCBI database. The gene, which was termed *VvXERICO*, is located on chromosome 12 and is intronless as well. The ORF, composed of 151 amino acids, showed 60% identity and 76% similarity to *XERICO*. Like XERICO, the VvXERICO protein has a highly conserved transmembrane motif (TM) and a RING-H2 motif (Ko et al., 2006).

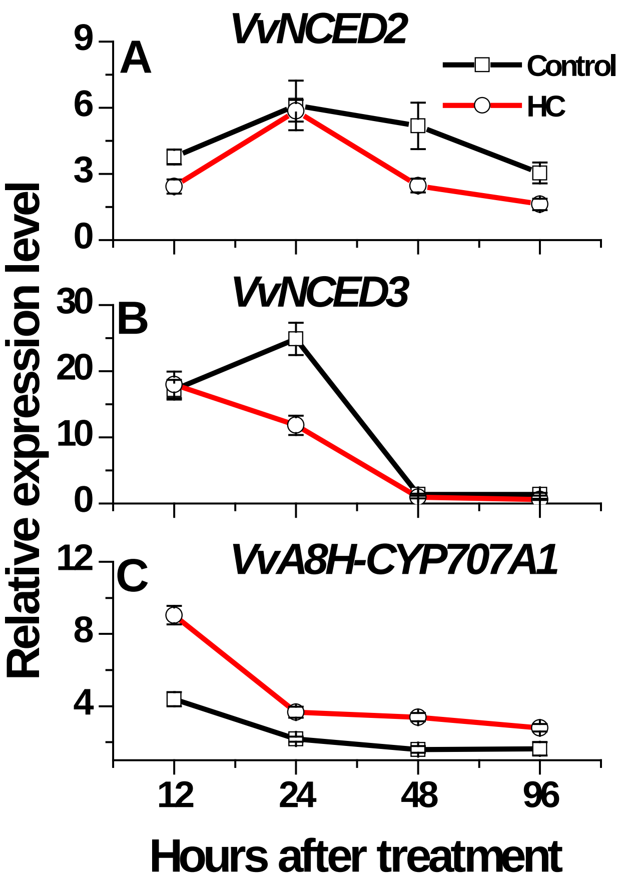

Supplementary Figure S3: Transcription modulation of additional bud-expressed *VvNCED* and *VvA8H-CYP707A* genes by HC. Transcript levels of *VvNCED2* (A), *VvNCED3* (B) and *VvA8H-CYP707A1* (C) were analyzed by qRT-PCR. Details are as described in Fig. 3.

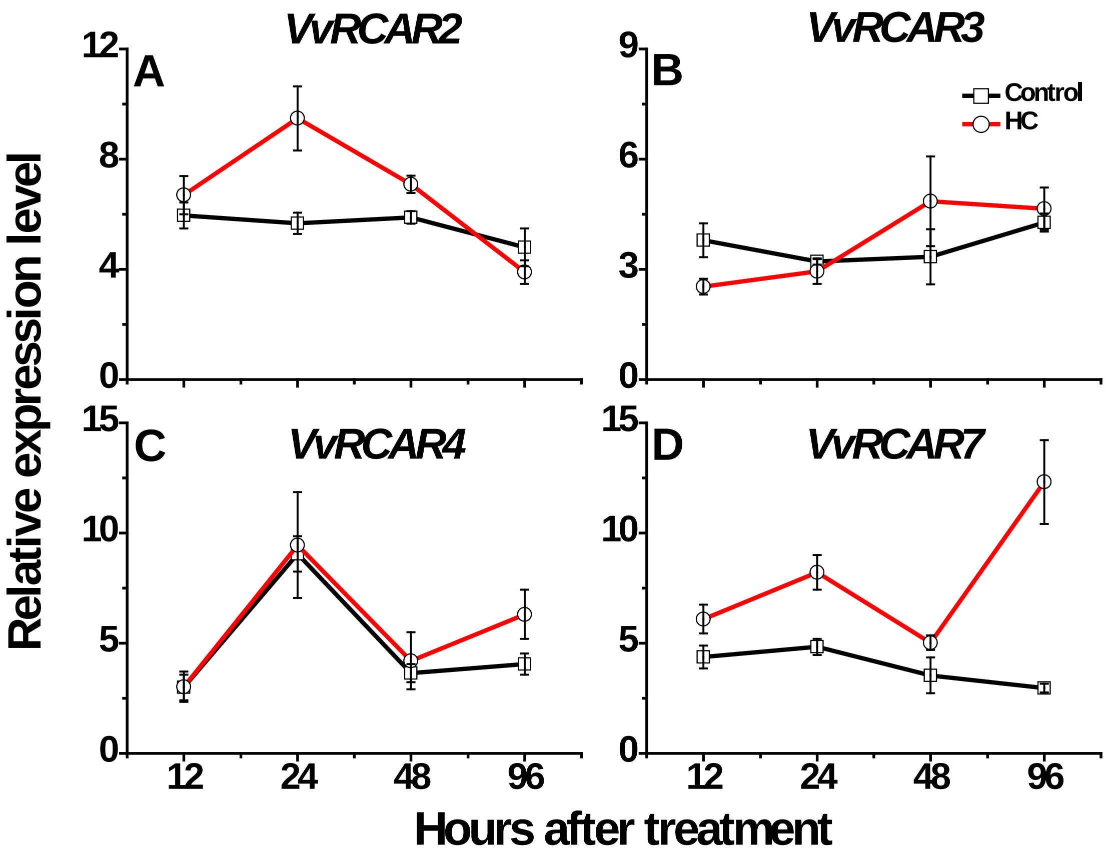

Supplementary Figure S4: Transcription modulation of additional bud-expressed *VvRCAR*s by HC. Transcript levels of *VvRCAR2* (A), *VvRCAR3* (B), *VvRCAR4* (C) and *VvRCAR7* (D) were analyzed by qRT-PCR. Details are as described in Fig. 3.

Supplementary Table S1: Primers used for gene expression analyses by qRT-PCR

	Forward primer (5' to 3')	Reverse primer (5' to 3')	Accession number
VvNCED1	GGGTGGTTGATGAGTATAGTGAG	CAACAAAAGTCCCATGAAAGCC	VV05G09670[a]
VvNCED2	GGATTTCTCCTGGACTCTGG	ACCGACAGGCAAAAGCTACAAG	VV10G04440[a]
VvNCED3	TTCCCTCACGAGTTCCCTATG	TCCTCTGCAATCTGACACCAAG	VV19G11960[a]
VvA8H-CYP707A1	GGTTGCTCCAAAGCCCAATAC	TGCCCACCATAGACCACCTG	VV02G09270[a]
VvA8H-CYP707A4	CTTGTCCTGGAAATGAGC	TGAGGAACAGGGAATGGTC	VV03G06660[a]
VvXerico	TGGTAAGCAATATCTGGGAGA	AGGCATAAAACTGGAGCAT	CAN82068.1[b]
VvRCAR1	TGATGGGAGACCAGGGACAC	TTTGAGGTTGCAGTTGATGAG	GSVIVT01027078001[c]
VvRCAR2	GGATGTGAAAGTGGGAATGG	GGAAGATGGAAGAAGGGCTAC	GSVIVT01028704001[c]
VvRCAR3	GGCAAAGCATTTGAGGAAC	GGGTAGCATTGAAAGGAAGAG	GSVIVT01019517001[c]
VvRCAR4	GCCAATTATCACTCGACTCTTAC	GCCTTGAGGTTGAACCCTATTATG	GSVIVT00035869001[c]
VvRCAR5	CGTGGTCCTGGAATCCTATG	GACGGCGTATTGGGATGTG	GSVIVT00037390001[c]
VvRCAR6	CGGGTAACACGAAGGAGGA	GGGGTGCAATAATCTAAAAGAG	GSVIVT01032747001[c]
VvRCAR7	AGAGTGGGATGCCTCAGAGA	CTCCCCTCCGATGATACTGA	GSVIVT01013161001[c]
VvPP2C2	GGCATCGAGTTTTTGGTGTT	TTGCCCGAGGAATAAATGTC	GSVIVT01035420001[c]
VvPP2C4	TGGGCTTTGGGATGTTATGT	TGTGCAGGAGTCTCATCAGC	GSVIVT01015308001[c]
VvPP2C9	TTAAAGCCCTTCGTGAGCTG	GACACCACGTCCCACAGAC	GSVIVT01024875001[c]
VvABF1	GGATACATCTTCCGTTTCAC	ATAAGCCTGTTTACGAGCC	XP_002284791[b]
VvABF2	GCCATGATTGGTTTGGGAACT	ACATAAGGAGCAGGCGATAAT	CAN64991[b]

[a]Accessions from PLAZA version 2: http://bioinformatics.psb.ugent.be/plaza/

[b]NCBI accessions

[c]Accessions from Grape Genome Browser: http://www.genoscope.cns.fr/externe/GenomeBrowser/Vitis/

Supplementary Table S2: Parameters for LC-ESI-MS/MS analysis

Method No.	Column	Solvent A	Solvent B	Composition of solvent B		
1	XDB-C18	Water containing 0.01% acetic acid	Acetonitrile containing 0.05% acetic acid	3 to 22% B over 27.5 min		
2	XDB-C18	Water containing 0.01% acetic acid	Acetonitrile containing 0.05% acetic acid	3 to 13% B over 15 min		
Hormone	LC method No.	Retention time (min)*	Charge	MS/MS (*m/z*)	Collision energy (V)	Fragmentor (V)
ABA	1	27.1	–	263/153	8	140
D_6-ABA	1	27.1	–	269/159	8	140
PA	1	18.3	–	279/139	10	130
D_3-DPA	1	18.3	–	282/142	10	130
DPA	1	11.4	–	281/171	16	140
D_3-DPA	1	11.4	–	284/174	16	140
neoPA	1	23.8	–	279/205	10	130
D_3-neoPA	1	23.8	–	282/208	10	130
ABA-GE	2	13.6	–	425/263	10	160
D_5-ABA-GE	2	13.6	–	430/268	10	160

*Retention time of deuterium-labeled internal standards were slightly shorter than non-labeled endogenous compounds.
